# Design of Intelligent Monitoring System in Galloping Power Transmission Line

**DOI:** 10.3390/s22114197

**Published:** 2022-05-31

**Authors:** Lijun Wang, Hao Li, Xu Lu, Xiangyang Li, Jianyong Zhang, Xinxin Wang, Changxin Chen

**Affiliations:** 1 School of Mechanical Engineering, North China University of Water Resources and Electric Power, Zhengzhou 450045, China; wanglijun@ncwu.edu.cn (L.W.); z20201040497@stu.ncwu.edu.cn (H.L.); 201608706@stu.ncwu.edu.cn (X.L.); wangxinxin@ncwu.edu.cn (X.W.); 2 School of Management and Economics, North China University of Water Resources and Electric Power, Zhengzhou 450045, China; z201910413352@stu.ncwu.edu.cn; 3 School of Computing, Engineering & Digital Technologies, Teesside University, Middlesbrough TS1 3BA, UK; j.zhang@tees.ac.uk; 4 School of Electrical and Control Engineering, North University of China, Taiyuan 030051, China

**Keywords:** transmission lines, Kalman filtering, Mahony complementary filter, galloping amplitude monitoring

## Abstract

To prevent the frequent occurrence of transmission line galloping accidents, many scholars have carried out studies. However, there are still many difficulties that have not been solved. To address the issues that have arisen during the installation of the monitoring system, a new installation technique for the galloping monitoring terminal structure has been developed, and structural design and transmission line impact have been taken into account. A method combining Kalman and Mahony complementary filtering has been shown to solve the problem of wire twisting when galloping is taken into account. The displacement is derived by double-integrating the acceleration, although the trend term has a significant impact on the integration result. To handle the trend term issue and other error effects, a method combining the least-squares method, the adaptive smoothing method, and the time-frequency domain hybrid integration approach is used. Finally, the monitoring terminal’s structural design is simulated and evaluated, and the measured amplitude is assessed on a galloping standard test bench. The difference between the measured amplitude and the laboratory standard value is less than 10%, meeting the engineering design criteria. And the galloping trajectory is identical to the test bench trajectory, which is critical for user end monitoring.

## 1. Introduction

With the state’s development of transmission line building size, the region covered by the power grid has grown in recent years, and the terrain traversed has become more complicated. Furthermore, because of the frequent occurrence of adverse weather, transmission line galloping accidents [1] are widespread. The galloping issue not only costs grid operators a lot of money, but also affects the satisfaction of microgrid operators and prosumers [2]. Transmission line galloping is characterized by the self-excited oscillation of low frequency (about 0.1–3 Hz) [3] and large amplitude (about 5–300 times of conductor line diameter). This phenomenon will result in significant accidents [4], such as tower falling, wire breakage, line fittings breaking, and line power failure tripping, which would severely disrupt the power grid’s functioning and result in incalculable economic loss. The eccentric icing phenomena of the conductor is what causes the transmission line to gallop. The unstable pneumatic lift force which is surrounding the conductor causes the wire to oscillate when subjected to wind stimulation.

Scholars in the United States and overseas have conducted extensive experimental and theoretical studies on transmission line galloping amplitude monitoring. Simultaneously, related researchers have presented their wire galloping amplitude monitoring systems and monitoring methods.

Huang et al. [5] proposed using gyro sensors and acceleration sensors to monitor the galloping of transmission lines and using Zigbee technology to transmit the collected data wirelessly to realize on the-line monitoring of transmission lines. Zhao et al. [6] proposed that wireless sensor technology can be used to monitor the amplitude of galloping by the acceleration sensor, and empirical mode decomposition can be used to get more accurate results to eliminate errors. Rui et al. [7] proposed using the strain effect of fiber grating to make a two-dimensional acceleration sensor that can solve the problems of substantial electromagnetic interference and power supply in transmission lines. Although they can measure the galloping amplitude data, there are certain problems. The twisting of the wire is not taken into account in the galloping monitoring system’s algorithm design, resulting in a significant deviation in the measured acceleration, which leads to inaccurate galloping amplitude data measurement.

Bjerkan et al. [8] proposed the measurement of the wire galloping using optical fiber sensors under high voltage. Although the monitoring equipment can also collect the corresponding data, the monitoring system is limited by selecting the installation location and the quantity of the equipment. At the same time, these conditions seriously restrict the accuracy of the data measurement of the monitoring system. Simultaneously, the installation of the equipment also faces the problem of installation difficulties. Ma et al. [9] proposed making use of a differential GPS positioning system to measure the galloping amplitude of the wire. GPS precision has a major influence on the accuracy of this monitoring system. The monocular vision was presented by Yang et al. [10] to monitor the galloping amplitude of transmission line conductors. The galloping amplitude of the transmission line may be calculated using the pixel change curve by following feature points in the galloping video. Li et al. [11] proposed an image of processing system to explain the automatic wire positioning and the clapboard identification, accurately displaying the wire height and clapboard status. The accuracy of the monitoring system is strongly affected by elements such as the camera’s installation position, the selection of galloping feature spots, and the angle between the camera and the wire.

Huang et al. [12] proposed a wireless sensor module to measure the magnitude of conductor galloping using acceleration and gyro sensors. This method’s algorithm design ignores the wire twist and the gyroscope’s zero-drift problem, resulting in inaccurate attitude angle calculation and, ultimately, the gravitational acceleration component in the measured acceleration not being completely filtered out. The structure design of the system is independent of the structure from the original transmission line. Also, the impact of the structure on the transmission line has not been taken into account when evaluating the system installation.

To eliminate the signal trend item, low-pass filtering, polynomial fitting, and mean value methods are used. Only high-frequency noise can be filtered out using the low-pass filtering approach. Although the polynomial fitting method is straightforward, the type of trend item must be specified ahead of time. The mean approach is straightforward, but it needs a significant amount of computation. Zhang et al. [13] proposed that the trend term is eliminated by using the windowed recursive least squares technique, which is applied to the measurement of polished rod movement in oil wells. Because of the difference in the number of window points, this method will have an effect on the findings. Zhang et al. [14] proposed using a wavelet method and empirical mode decomposition to extract the signal’s trend term. In this approach, the EMD decomposition necessitates some previous knowledge, and the decomposition is influenced by the end-point oscillation. Li et al. [15] proposed to use the moving average method to extract the trend term in the MEMS gyro signal. This approach is straightforward, but it necessitates several comparisons of the smoothing order and smoothing times. Cheng et al. [16] proposed wavelet denoising and time-frequency integration methods to obtain displacement signals. To acquire better results, the signal must be preprocessed before time-frequency integration. As a result, time-frequency domain hybrid integration, smooth filtering, and the least-squares approach are presented as the best ways to restore transmission line galloping amplitude information.

To summarize the issues with the wire galloping monitoring system, they are as follows: No consideration was given to the installation of monitoring systems and the impact on the original transmission line in the design of the wire galloping monitoring system. The torsion phenomena generated by the wire galloping was ignored when the galloping amplitude algorithm was built. Although acceleration integration yields a bigger trend term, it is unable to effectively eliminate the trend term.

This paper’s contribution to the above issues is as follows: (1) Aiming at the problematic installation of the sensor monitoring system, combining the spacer bar with the galloping monitoring system was proposed to design the three-dimensional structure of the four-split spacer bar. The improved three-dimensional structure was subjected to a two-way fluid-structure coupling analysis, and the effect of the improved structure on wire galloping amplitude was assessed; (2) Given the problem that the twisting of the wire itself is not considered in the algorithm design, this paper proposes to use the Kalman filter and the Mahony complementary filtering algorithm to solve the galloping posture of the wire. The attitude calculation solves the problem of inaccurate acceleration measurement caused by the twist of the wire itself when the wire is galloping; and (3) To address the significant trend term induced by the double integration in the time domain, an improved smoothing filtering technique and the time-frequency domain hybrid integration approach are proposed.

## 2. Design Method of Galloping Amplitude Monitoring System

### 2.1. Principle of Galloping Amplitude Monitoring System

A si*x*-axis attitude sensor is presented as a terminal sensor for wire galloping amplitude monitoring. The basic principle of wire galloping is that the three-axis acceleration sensor in the si*x*-axis attitude sensor monitors the acceleration signals in the x, y, and z axes. The acceleration is double-integrated in the time domain to generate the displacement signal. Take, for example, the *y*-axis direction.

Velocity and displacement are calculated with the following formula:

(1) $$v_{y}\left(t\right)-v_{y}\left(t_{0}\right)=\int_{t_{0}}^{t}a_{x}\left(t\right)dt,$$

(2) $$s_{y}\left(t\right)-s_{y}\left(t_{0}\right)=\int_{t_{0}}^{t}v_{y}\left(t\right)dt,$$

Formula: $a_{y}\left(t\right)$ is the acceleration at time *t* (m/s^2^); $v_{y}\left(t\right)$ is the velocity at time *t* (m/s); and $s_{y}\left(t\right)$ is the displacement at time *t* (m).

### 2.2. Galloping Monitoring Terminal Design

**Figure 1:**
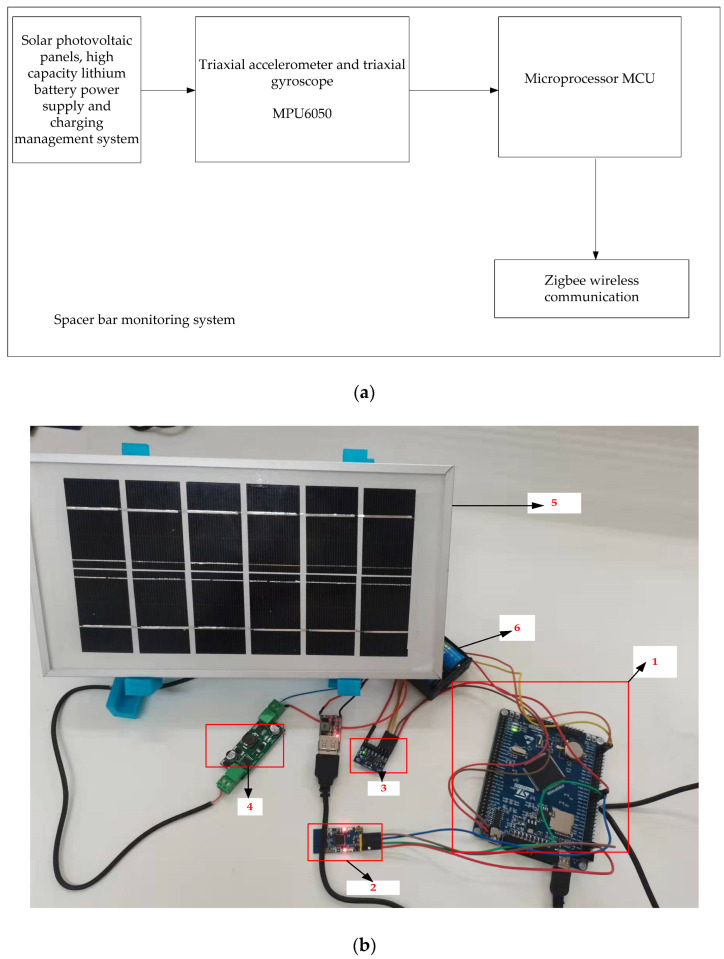
Structural block diagram and physical drawing of the on-line monitoring system. (**a**) Structural block diagram of the on-line monitoring system; (**b**) Physical map of the on-line monitoring system.

The structural properties of high-voltage transmission lines are used to design a wire gallop amplitude monitoring system. A si*x*-axis attitude sensor, an MCU, a wireless communication module, and a power management system make up the on-line galloping monitoring system. Figure 1 depicts the on-line monitoring system’s structural block diagram and physical map. An STM32F103 microcontroller, a MEMS inertial sensor MPU6050 (3-axis acceleration, 3-axis gyroscope), solar photovoltaic panels, a power management system, and a wireless communication module make up the on-line monitoring system (Zigbee module [17,18,19]). The si*x*-axis attitude sensor in the galloping monitoring system monitors the wire’s galloping acceleration in three dimensions and the torsion angle in three directions. The wire galloping acceleration data may be gathered in real-time by conducting attitude calculations on the acceleration and angular velocity using Mahony complementary filtering. We can acquire the wire’s galloping amplitude data by processing the acceleration data, and the wire’s galloping trajectory curve may be fitted using the galloping amplitude data.

The descriptions of each device in Figure 1b are as follows: 1 is the minimum system of single-chip microcomputers, and its primary function is that the algorithm control core of the whole system controls the work of each piece of equipment in the entire monitoring system and data processes; 2 is the Zigbee module, the primary function is to send the collected data wirelessly; 3 is the attitude sensor module, whose primary function is to monitor the acceleration and angular velocity of the wire galloping in real-time; 4 is the lithium battery charging management module, whose primary part is to protect the safety of the lithium battery when the solar photovoltaic panel charges the lithium battery; 5 is a solar photovoltaic panel, the primary function of which is to convert solar energy into electrical energy to charge the lithium battery; and 6 is a high-capacity lithium battery whose primary part is to supply power to the entire monitoring system.

### 2.3. Design of Power Management System

**Figure 2:**
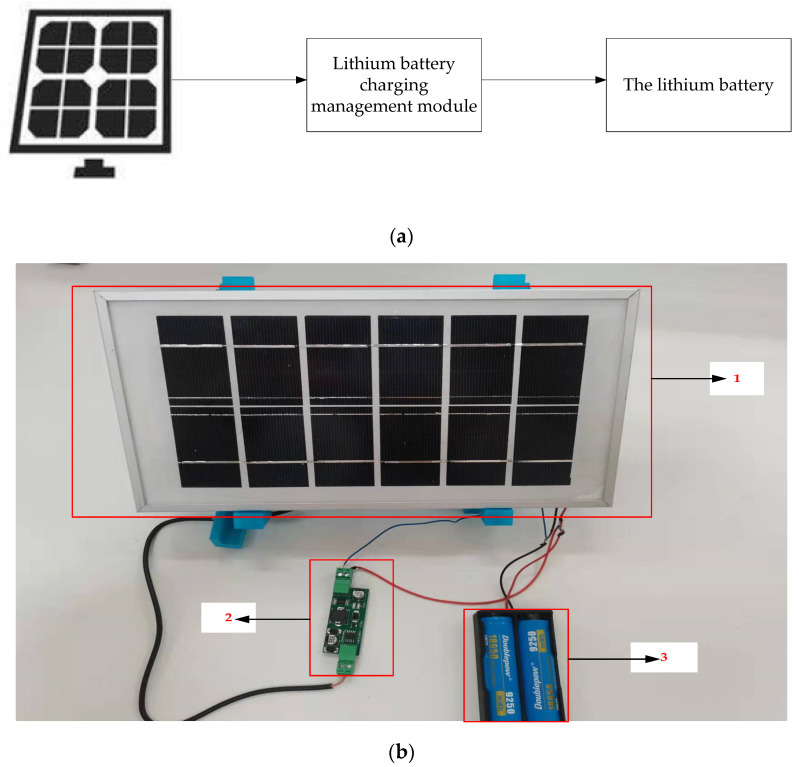
Structure block diagram and physical drawing of the power management system. (**a**) Block diagram of power management system structure; (**b**) Physical map of the power management system.

The terminal of the galloping monitoring system needs to be fixed on the high-voltage transmission line to minimize the size and weight of the system. Because the terminal node must fulfill long-term operational requirements, the power management system is built with solar photovoltaic panels, large-capacity lithium batteries, and lithium battery charging management modules. The block diagram and physical map of the power management system are shown in Figure 2. The power management system’s solar PV panel is monocrystalline silicon, and the lithium battery management module is a CN3705 module that can safeguard lithium batteries from overcharging and over-discharging. To enhance the charging safety of lithium batteries, the lithium battery management module is separated into three charging modes: constant current, constant voltage, and trickle. The system is powered by lithium batteries with a high energy density.

The descriptions of each device in Figure 2b are as follows: 1 is a solar photovoltaic panel. Its primary function is to convert solar energy into electrical energy to charge the lithium battery; 2 is the lithium battery charging management module. Its primary part is to protect the safety of the lithium battery when the solar photovoltaic panel charges the lithium battery; and 3 is a high-capacity lithium battery whose main function is to supply power to the entire monitoring system.

### 2.4. Installation Method of Galloping Monitoring System Terminal

#### 2.4.1. Terminal Installation Design

**Figure 3:**
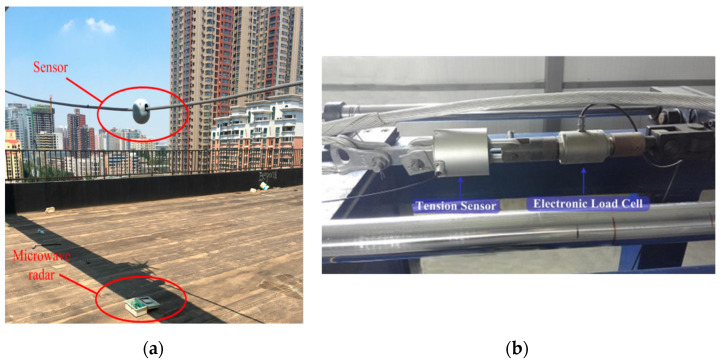
Installation mode of original monitoring system. (**a**) The monitoring terminal is installed on the wire [12]; (**b**) The sensor is mounted on the wire [20].

**Figure 4:**
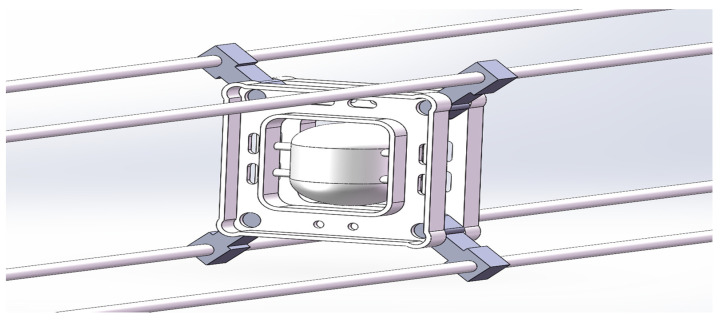
3D structure diagram of improved spacer.

The galloping amplitude monitoring system’s terminal must be mounted directly on the transmission line. The structural design of the galloping monitoring terminal in this article takes into account the monitoring system’s climatic characteristics, the terminal node’s post-maintenance, and the limitations of the traditional terminal node installation technique. The terminal nodes of a conventional transmission line galloping monitoring system are installed directly on the transmission lines. Figure 3 depicts the conventional monitoring system installation approach. The installation method of this kind of galloping monitoring equipment is challenging to install, and it also brings considerable difficulties in the post-maintenance of the sensor. A better design of the spacer bar device is presented, taking into account the effect of different elements. The spacer structure has been improved to enable the installation of the galloping monitoring system terminal on the spacer, which not only addresses the terminal installation difficulty but also enables the intelligent spacer function while maintaining the original spacer’s functional features. Figure 4 depicts the 3D structural diagram of the improved spacer bar.

#### 2.4.2. Analysis of Influence of Monitoring Terminal on Conductor Galloping

The galloping monitoring system’s terminal is situated on a high-voltage transmission line, and improvements to the spacer bar construction will influence wire galloping. After a large number of simulated trials, the influence of the improved spacer bar structure and the original spacer bar structure in the wind field was investigated. The influence of the improved spacer bar on the original transmission line is intuitively measured using the galloping displacement simulation data.

**Figure 5:**
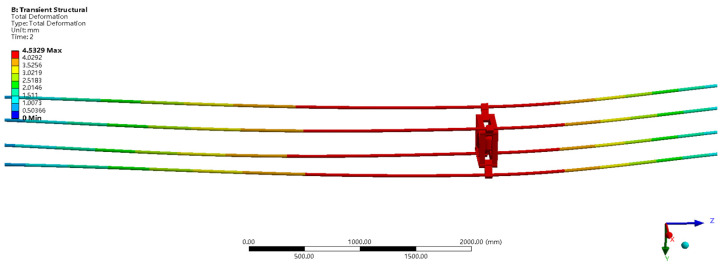
A cloud map showing the improved spacer bar’s galloping displacement.

**Figure 6:**
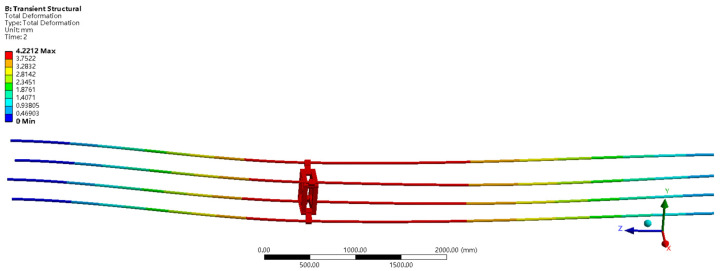
The initial galloping displacement cloud map of the original spacer bar.

The spacer bar and wire are then formed into a structure. In the wind field, a two-way fluid-structure [21] interaction simulation study was done using Ansys Workbench software [22,23] on the original spacer bar and the improved spacer bar. According to available data, the angle between the wind direction and the conductor cross-section determines the amplitude of the conductor galloping. Under the same wind speed, when the angle between the cross-section of the wire and the wind direction is 0°, the amplitude of the wire galloping is the largest. The original spacer bar and the improved spacer bar were placed in the same wind field environment [24] to analyze and research the impact of the improved spacer bar construction on the original transmission line. The simulation system in Fluent [25] is configured at a wind speed of 15 m/s, a wind direction parallels the wire cross-section, and is set to a turbulence model for the wind field. Because of the coupling effect between the transmission line and the air, the Fluent and transient structures have to be systematically connected in the simulation study. The wire’s galloping amplitude may be estimated using the Fluent-transient structure connection. The impact of the improved spacer bar on the original transmission line may be more intuitively analyzed using the galloping amplitude data of the wires. Figure 5 depicts the galloping displacement of the improved spacer bar structure. Figure 6 depicts the galloping displacement of the original spacer bar structure.

Under the same wind field circumstances, the maximum galloping displacements of the improved spacer bar construction and the original spacer bar structure are 4.5329 mm and 4.2212 mm, respectively, according to the wire galloping displacement data in Figure 5 and Figure 6. The galloping displacement of the improved spacer is more than that of the original spacer, as can be seen from the maximum galloping displacement; however, the galloping displacement difference is just 7.38 percent. The galloping displacement error data between the improved spacer bar and the original spacer bar is calculated using simulation, and it can be seen that the improved spacer bar’s structural design matches the genuine engineering needs.

## 3. Algorithm Design for Galloping Amplitude

The monitoring terminal of this intelligent galloping on-line monitoring system collects three-axis acceleration and three-axis gyroscope data from each galloping monitoring location using 3-axis acceleration and 3-axis gyroscope sensors, respectively. In principle, integrating the acceleration yields the velocity and displacement data of galloping. The straight double integration of the acceleration data will generate a more significant trend item, which can entirely drown the original galloping displacement data. At the same time, as the wire gallops, the component of gravity acceleration merges with the component of body acceleration, resulting in erroneous acceleration measurement. A novel solution is offered to the problems that exist in the current galloping monitoring system.

### 3.1. Algorithm Design for Galloping Attitude

**Figure 7:**
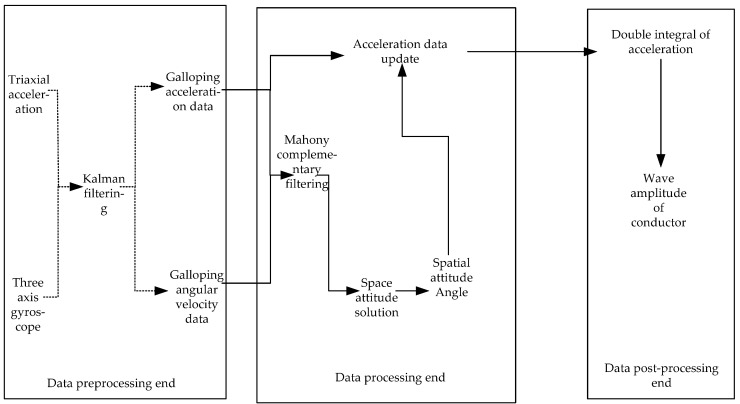
Schematic diagram of the design of the galloping attitude calculation algorithm.

The torsion [26,27] of the wire during the galloping process is unavoidable; therefore, the measured acceleration data and the acceleration data of the wire galloping are not in the same coordinate system. This is because the gyro sensor exhibits zero drift and noise interference will inevitably be introduced during the operation of the sensor. As a result, the Kalman filtering [28,29,30] and Mahony complementary filtering algorithms are given to tackle the twisting issue of the wire itself and the sensor’s noise interference. Figure 7 depicts the design schematic diagram of the galloping attitude calculating method.

### 3.2. Kalman Filter and Mahony Complementary Filter Are Fused

**Figure 8:**
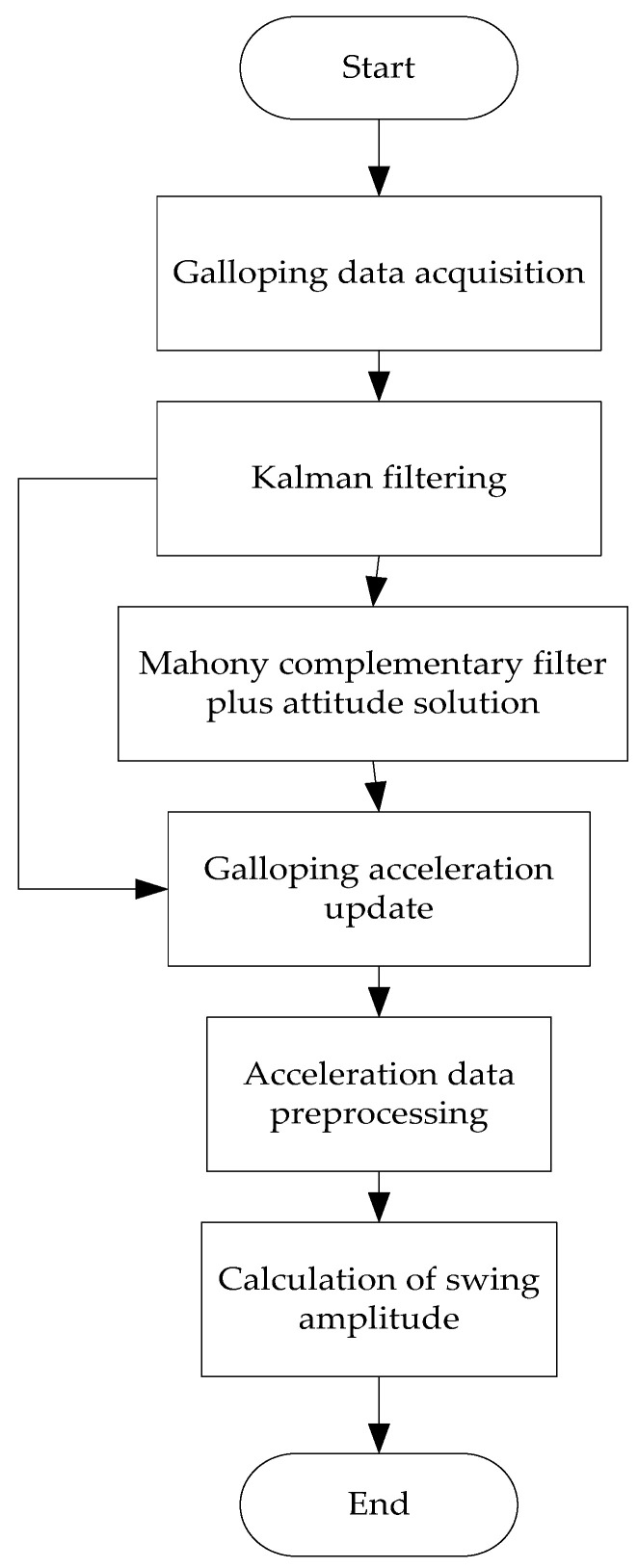
Algorithm Design Flowchart.

The design principle of the galloping attitude calculation algorithm is as follows: First, the raw data from the three-axis acceleration and gyroscope sensors are subjected to Kalman filtering to reduce noise interference. Second, the Mahony complementary filtering technique is used to process the acceleration and gyroscope data. The galloping attitude angle of the wire can be obtained by data fusion of the acceleration data and the gyroscope data. Filtering off the component of gravitational acceleration via the galloping attitude angle yields accurate acceleration data. Finally, the newest acceleration is preprocessed, and the galloping amplitude is calculated using time-frequency domain hybrid integration. The whole algorithm design flow chart is shown in Figure 8.

#### 3.2.1. Kalman Filter Algorithm Design

The Kalman filter is an efficient recursive filter that can estimate the state of a dynamic system from incomplete and noisy measurements. The Kalman filter just needs to know the estimated value of the previous instant to calculate the estimated value of the present state. Kalman is presented to anticipate the future state of the system and output acceleration and angular velocity data based on the signal characteristics of the data in this architecture.

A 3-axis acceleration and a 3-axis gyroscope make up the si*x*-axis attitude sensor. Noise and a slowly changing bias term frequently disrupt sensor results. The three-axis accelerometer measures the acceleration as:

(3) $$a_{n}=a_{l}+a_{i}+a_{m},\;$$

In the formula: $a_{n}$ is the measured acceleration value; $a_{i}\sim N(0,\delta_{a})$ is zero-mean noise; $a_{m}$ is the bias term that changes slowly; $a_{l}$ is the actual value of acceleration.

The angular velocity measured by the three-axis gyroscope is

(4) $$\omega_{n}=\omega_{l}+\omega_{i}+\omega_{m},$$

In the formula: $\omega_{n}$ is the measured angular velocity value; $\omega_{i}$ is zero-mean noise; $\omega_{m}$ is the bias term that changes slowly; $\omega_{l}$ is the actual value of angular velocity.

The data measured by the acceleration sensor is selected as the input, and the state variable equation is derived by the Kalman filter as follows:

(5) $$\left\{ \begin{array}{l} X_{k}=\Phi_{k/k-1}X_{k-1}+W_{k-1}+Q_{k-1}M_{k-1}\\ Z_{k}=H_{k}X_{k}+V_{k}+f\Phi_{k/k-1} \end{array}\right.$$

In the formula: $X_{k-1}$ is the system state equation; $W_{K/K-1}$ is system noise; $H_{K}$ is measurement matrix; $\Phi_{k/k-1}$ is state transition matrix; $V_{K}$ is measurement noise; $Q_{K-1}$ is the input coefficient matrix; $M_{k-1}$ is the acceleration state;

Assuming that the mean values of noise $W_{k-1}$ and $V_{k}$ are both 0, the estimated mean square error is:

(6) $$P=E[xx^{T}],$$

The state update equation is

(7) $$K_{K}=\frac{P_{K/K-1}H_{K}^{T}}{H_{K}P_{K/K-1}H_{K}^{T}+R_{K}},$$

(8) $${\hat{X}}_{K}={\hat{X}}_{k/k-1}+K_{k}(Z_{k}-H_{k}{\hat{X}}_{K/K-1})+Q_{k-1}{\hat{M}}_{k-1},$$

(9) $$P_{K}=(I-K_{K}H_{K})P_{K/K-1},$$

After Kalman filtering, the acceleration data is $a_{p}$, and angular velocity data is $\omega_{p}$.

#### 3.2.2. Mahony Complementary Filtering Algorithm Design

**Figure 9:**
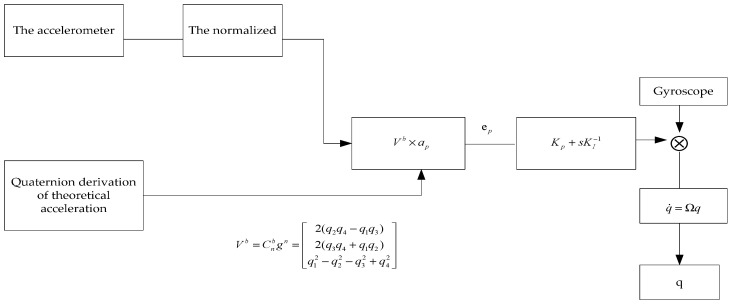
Mahony complementary filter schematic.

The following is the design idea of Mahony’s complementary filtering algorithm: the sensor’s measured acceleration is cross-multiplicated with the theoretical acceleration derived from the quaternion [31,32], and the error angle between the two vectors is calculated. The error angle between the two vectors is utilized to compensate for the mistake generated by the gyroscope’s zero-drift using the theoretical concept of PI control. Finally, the quaternion approach is used to solve the attitude angle. Figure 9 shows a schematic diagram of the Mahony complementary filtering method.

Galloping will cause the wire to twist, resulting in an angle between the coordinate system where the sensor is located and the geographic coordinate system. Therefore, the two coordinate systems need to be unified. The transformation of two coordinate systems requires a coordinate rotation matrix to achieve the unification between the two coordinate systems. The coordinate rotation matrix is:

(10) $$C_{n}^{b}=\left[\begin{array}{ccc} \cos ϒ\cos\alpha +\sin ϒ\sin\theta \sin\alpha & -\cos ϒ\sin\alpha +\sin ϒ\cos\alpha \sin\theta & -\sin ϒ\cos\theta \\ \sin\alpha \cos\theta & \cos\alpha \cos\theta & \sin\theta \\ \sin ϒ\cos\alpha -\cos ϒ\sin\alpha \sin\theta & -\sin ϒ\sin\alpha -\cos ϒ\cos\alpha \sin\theta & \cos ϒ\cos\theta \end{array}\right]$$

It can be seen from the coordinate rotation matrix that the Euler method has a large amount of calculation, and the conversion between quaternions and Euler angles is used to reduce the amount of calculation. When Euler angles are represented by quaternions, the direction cosine matrix may be calculated as follows:

(11) $$C_{n}^{b}=\left[\begin{array}{ccc} q_{1}^{2}+q_{2}^{2}-q_{3}^{2}-q_{4}^{2} & 2(q_{2}q_{3}+q_{1}q_{4}) & 2(q_{2}q_{4}-q_{1}q_{3})\\ 2(q_{2}q_{3}-q_{1}q_{4}) & q_{1}^{2}-q_{2}^{2}+q_{3}^{2}-q_{4}^{2} & 2(q_{3}q_{4}+q_{1}q_{2})\\ 2(q_{2}q_{4}+q_{1}q_{3}) & 2(q_{3}q_{4}-q_{1}q_{2}) & q_{1}^{2}-q_{2}^{2}-q_{3}^{2}+q_{4}^{2} \end{array}\right],\;$$

The gravitational acceleration in the geographic coordinate system is g, the expression is $g^{n}={\left[\begin{array}{ccc} 0 & 0 & 1 \end{array}\right]}^{T}$, will $g^{n}$ by converting the quaternion transformation matrix to the carrier coordinate system, in the carrier coordinate system, the gravitational acceleration matrix may be calculated as $V^{b}$.

(12) $$V^{b}=C_{n}^{b}g^{n}=\left[\begin{array}{c} 2(q_{2}q_{4}-q_{1}q_{3})\\ 2(q_{3}q_{4}+q_{1}q_{2})\\ q_{1}^{2}-q_{2}^{2}-q_{3}^{2}+q_{4}^{2} \end{array}\right]$$

After Kalman filtering, the acceleration data become $a_{px1},a_{py1},a_{pz1}$. Then, to get the normalized acceleration value, normalize the acceleration value:

(13) $$\left\{ \begin{array}{l} a_{px}=a_{px1}{\left(a_{px1}^{2}+a_{py1}^{2}+a_{pz1}^{2}\right)}^{-1/2}\\ a_{py}=a_{py1}{\left(a_{px1}^{2}+a_{py1}^{2}+a_{pz1}^{2}\right)}^{-1/2}\\ a_{pz}=a_{pz1}{\left(a_{px1}^{2}+a_{py1}^{2}+a_{pz1}^{2}\right)}^{-1/2} \end{array}\right.,\;$$

The error correction amount of the gyroscope may be calculated by multiplying the theoretical acceleration vector by the measured acceleration vector.

(14) $$e_{p}=V^{b}\times a_{p}=\left|\begin{array}{ccc} i & j & k\\ v_{x} & v_{y} & v_{z}\\ a_{px} & a_{py} & a_{pz} \end{array}\right|=\left|\begin{array}{c} v_{y}a_{pz}-v_{z}a_{py}\\ -v_{x}a_{pz}+v_{z}a_{px}\\ v_{x}a_{py}-v_{y}a_{px} \end{array}\right|$$

Gyroscope total error compensation e.

(15) $$e=e_{p}=\left[\begin{array}{c} e_{x}\\ e_{y}\\ e_{z} \end{array}\right]=\left|\begin{array}{c} v_{y}a_{pz}-v_{z}a_{py}\\ v_{z}a_{px}-v_{x}a_{pz}\\ v_{x}a_{py}-v_{y}a_{px} \end{array}\right|$$

For gyroscope compensation, error compensation using PI control is as follows:

(16) $$e^{\prime }(\omega )=K_{p}e(\omega )+\int_{0}^{t}K_{I}e(\omega )d\omega$$

The angular velocities after error compensation are respectively $\omega_{px}^{b},\omega_{py}^{b},\omega_{pz}^{b}$, then the differential equation of quaternion is:

(17) $$\left[\begin{array}{c} {\dot{q}}_{1}\\ {\dot{q}}_{2}\\ {\dot{q}}_{3}\\ {\dot{q}}_{4} \end{array}\right]=\left[\begin{array}{cccc} 0 & -\omega_{px}^{b} & -\omega_{py}^{b} & -\omega_{pz}^{b}\\ \omega_{px}^{b} & 0 & \omega_{pz}^{b} & -\omega_{py}^{b}\\ \omega_{py}^{b} & -\omega_{pz}^{b} & 0 & \omega_{px}^{b}\\ \omega_{pz}^{b} & \omega_{py}^{b} & -\omega_{px}^{b} & 0 \end{array}\right]\left[\begin{array}{c} q_{1}\\ q_{2}\\ q_{3}\\ q_{4} \end{array}\right]$$

Using the quaternion to solve the attitude angle of the wire galloping is:

(18) $$\left\{ \begin{array}{l} \alpha =-\arctan(2(q_{2}q_{4}-q_{1}q_{3}){\left(q_{1}^{2}-q_{2}^{2}+q_{3}^{2}-q_{4}^{2}\right)}^{1/2})\\ \theta =\arcsin(2(q_{1}q_{2}+q_{3}q_{4}))\\ \gamma =-\arctan(2(q_{2}q_{4}-q_{1}q_{2}){\left(q_{1}^{2}-q_{2}^{2}-q_{3}^{2}+q_{4}^{2}\right)}^{1/2}) \end{array}\right.$$

The combined algorithm of Kalman filtering and Mahony complementary filtering is compared with the attitude angle measured by the quaternion attitude solution alone. The galloping amplitude monitoring system’s attitude sensor is mounted on the turntable. The turntable is rotated at a set angle each time to compare the inaccuracies between the measured values of the two methods and the actual input values. It is more accurate to evaluate which algorithm is based on the error angle. The three coordinate axes of the attitude sensor are given 0°, 30°, 60°, and 90° inputs, respectively, on the turntable, and the accuracy of the system is evaluated by the measured values of the attitude angle. The above steps are repeated to process the attitude angles monitored by the two algorithms. The test results are shown in Table 1.

The combination of the Kalman filter and the Mahony complementary filter is closer to the actual input value than the attitude angle measured by the quaternion algorithm alone according to the experimental findings in the table. The results of the experiments demonstrate that the attitude angle method proposed in this research has a tiny inaccuracy with the real input value. A more precise attitude angle can reduce the influence of gravitational acceleration.

#### 3.2.3. Algorithm Design of Filtering out Gravitational Acceleration Component

The impact of gravitational acceleration must be filtered out since the wire galloping causes gravity to create the gravitational acceleration component on the carrier coordinate system. Subtract the gravitational acceleration component on each axis from the sensor’s measured acceleration. The problem of erroneous acceleration measurement caused by wire twisting can be solved by this method.

The components of gravitational acceleration on each axis may be computed using the coordinate rotation matrix. Euler angles are used to describe the gravitational acceleration component of each axis:

(19) $$g=\left[\begin{array}{c} g_{x}^{b}\\ g_{y}^{b}\\ g_{z}^{b} \end{array}\right]=C_{n}^{b}\left[\begin{array}{c} 0\\ 0\\ g \end{array}\right]=\left[\begin{array}{c} -g\sin\gamma \cos\theta \\ g\sin\theta \\ g\cos\lambda \cos\theta \end{array}\right]$$

More accurate acceleration data can be produced by subtracting the gravitational acceleration component from the acceleration processed by the Kalman filter. The latest acceleration calculation formula is derived as follows:

(20) $$\left\{ \begin{array}{l} a_{x}=a_{px1}-g_{x}^{b}=a_{px1}+g\sin\gamma \cos\theta \\ a_{y}=a_{py1}-g_{y}^{b}=a_{py1}-g\sin\theta \\ a_{z}=a_{pz1}-g_{z}^{b}=a_{pz1}-g\cos\lambda \cos\theta \end{array}\right.$$

### 3.3. Acceleration Data Preprocessing

Noise interference will inevitably be introduced in the functioning of the galloping monitoring system sensor due to the sensor’s complicated operating environment. If additional interfering signals are present in the acceleration data acquired by the accelerometer, the double integration in the time domain produces bigger trend components. The true signal will be fully drowned as a result of these trend elements. The least-squares approach, adaptive smoothing filtering algorithm, and time-frequency domain hybrid integration are used to tackle this problem.

#### 3.3.1. Least Squares Detrend Term

The time between any two consecutive data points may be estimated using the characteristics of the galloping amplitude monitoring system, assuming the digital signal captured by the galloping amplitude monitoring system is $\left\{x_{k}\right\}$ (*k* = 1, 2, 3, …, *n*). An *m*-th order polynomial is used to fit the acceleration signal, and the polynomial is as follows:

(21) $${\hat{x}}_{k}=a_{0}+a_{1}k+a_{2}k^{2}+\ldots +a_{m}k^{m}(k=1,2,3,\ldots ,n)$$

To better fit the *m*-th order polynomial to the acceleration signal, the following polynomial conditions must be met:

(22) $$E=\sum_{k=1}^{n}{\left({\hat{x}}_{k}-x_{k}\right)}^{2}$$

(23) $$\frac{\partial E}{\partial a_{i}}=0(i=0,1,2,\ldots ,m)$$

(24) $$\sum_{k=1}^{n}\sum_{j=0}^{m}a_{j}k^{j+1}-\sum_{k=1}^{n}x_{k}k^{i}=0(i=0,1,2,\ldots ,m)$$

From the conditions of the above formula, *m* + 1 undetermined coefficients $a_{j}$ can be derived.

**Figure 10:**
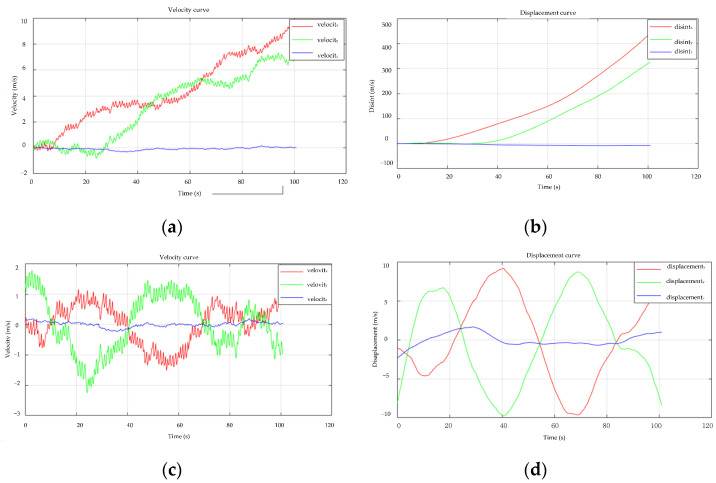
Data curve before and after least-squares method. (**a**) Unprocessed speed curve; (**b**) Displacement curve obtained without processing; (**c**) After processing, the speed curve is obtained; (**d**) Displacement curve obtained after processing.

Figure 10 shows the data integration curve before and after the trend term is eliminated using least-squares fitting. Using least-squares fitting to eliminate the trend component can better decrease the effect of interference in the signal, whether it is the velocity acquired by single integration or the displacement obtained by double integration, as can be seen from the data integration curve.

#### 3.3.2. Design of Adaptive Smoothing Filtering Algorithm

Periodic errors will inevitably arise during data integration, resulting in spikes and burrs on the integral data curve. Smoothing filtering is essential for processing to eliminate these mistakes from the data curve. When smoothing filters are used, the smoothing order is an important indicator in the smoothing filtering process. The following circumstances will arise due to the smoothing order difference: The data is severely deformed when the smoothing order is large. At the same time, the smoothing effect on the data curve is poor when the smoothing order is low. As a result, to acquire more accurate galloping amplitude data, the appropriate smoothing order must be determined. When the wire’s cross-section is the same, taking into account the correlation between wind speed and galloping acceleration has a substantial influence on the accuracy of the final galloping amplitude. The following is the best smoothing order correlation expression:

(25) $$r\left(v_{i},a_{i}\right)=\frac{\sum v_{i}a_{i}-n\bar{va}}{(n-1)s_{v}s_{a}}=\frac{n\sum v_{i}a_{i}-\sum v_{i}\sum a_{i}}{\sqrt{(n\sum v_{i}^{2}-(\sum v_{i}{)}^{2})\left(n\sum a_{i}^{2}-{\left(\sum a_{i}\right)}^{2}\right)}},$$

(26) $$\left|r\left(v_{i},a_{i}\right)\right|\leq 1,$$

The formula: r is the Pearson correlation coefficient [33]; $v_{i}$ is the wind speed; $a_{i}$ is the acceleration; *n* is the number of sampling points.

**Figure 11:**
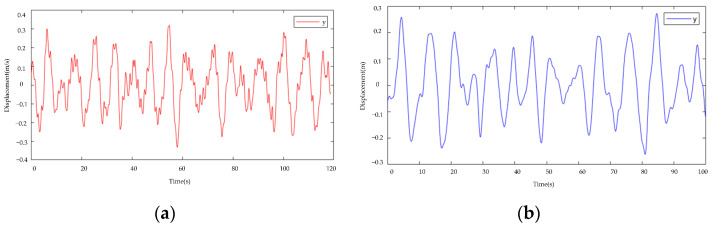
Data curve before and after smoothing filtering. (**a**) Data curves are not processed with smoothing filtering; (**b**) Smooth filter processing data curve.

The integral data curve after smoothing and the integral data curve without smoothing is shown in Figure 11. It can be observed from the data curves of Figure 11a,b that the integrated data curve is smoother after smoothing filtering, whereas the integrated curve without smoothing filtering contains pulse peaks.

### 3.4. Galloping Amplitude Algorithm

The error caused by single integration in the time domain is minor due to the significant accumulated error in the process of double integration in the time domain. The error will be reduced by using time-domain single integration, the least-squares approach to eliminate the trend component, and smooth filtering. The low-frequency signal has a greater impact on the double integral in the frequency domain, but the low-frequency signal has a lesser influence on the single integral in the frequency domain. As a result, the time-frequency domain hybrid integration algorithm is proposed, which combines the benefits of time-domain and frequency-domain integration. The velocity is obtained by first integrating the processed acceleration data in the time domain and removing the trend term. Then, using the Fourier transform, the speed is transferred to the frequency domain and one integration in the frequency domain is undertaken. Lastly, inverse Fourier transform is used to obtain the galloping amplitude in the time domain.

**Figure 12:**
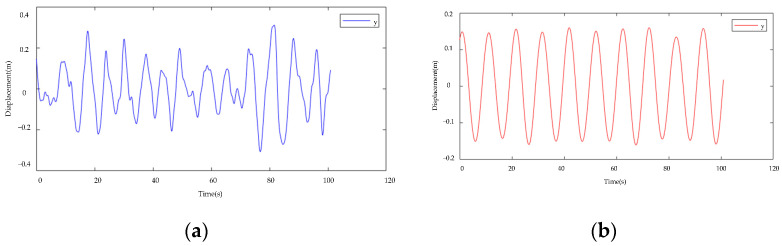
Different integration techniques’ data curves. (**a**) Double Integral Curve in Time Domain; (**b**) Time-frequency domain hybrid integration curve.

The data curves of the double integration in the time domain and the time-frequency domain hybrid integration algorithm are shown in Figure 12. Compare the integration curve obtained by the double integration in the time domain with the integration curve obtained by the time-frequency domain hybrid integration. The original input signal is an equal-amplitude vibration signal. The integrated data curve in the figure shows that the time-frequency domain hybrid integration algorithm is superior to the amplitude data acquired by double integration in the time domain.

## 4. Analysis of the Wire Galloping Experiment

### 4.1. Wire Galloping Experiment Setup

The conductor galloping experiment was conducted on the transmission line galloping test bench provided by the State Grid Transmission Line Galloping Prevention and Control Laboratory. The experimental platform serves as a base for assessing if any galloping monitoring system is accurate. The wire galloping is produced by the mechanical vibration of the galloping test bench. Change the galloping amplitude of the wire by a given input signal.

**Figure 13:**
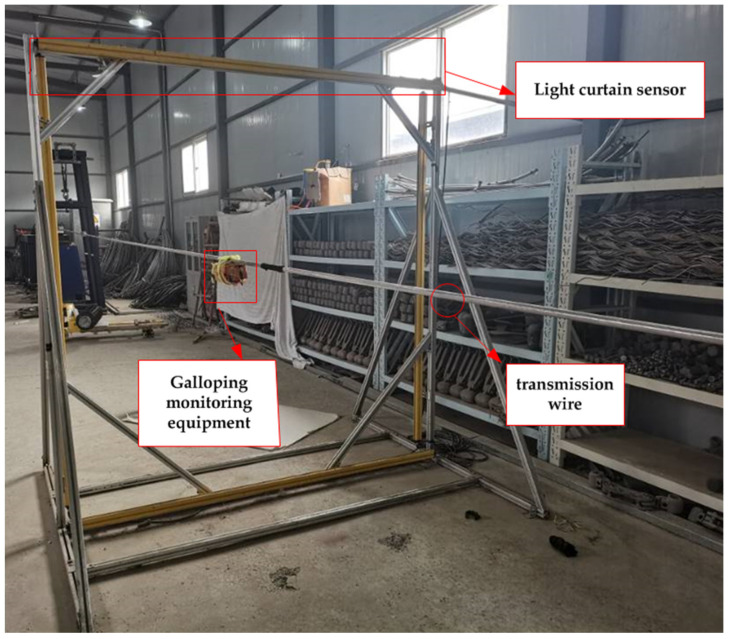
Conductor galloping experimental equipment.

The prototype of the galloping monitoring system was mounted on the wire to test the accuracy of the designed galloping monitoring system’s amplitude measurement. The light curtain sensor measures the test bench’s galloping amplitude. When the wire begins to gallop, the light curtain sensor checks the wire’s position in real-time using the light emitted to get the test bench’s galloping amplitude data. Give the test bench specific input values during the experiment. Allow the galloping monitoring system to begin collecting the galloping acceleration when the test bench’s monitoring terminal detects that the galloping amplitude has reached the goal amplitude and there is galloping with the same amplitude. When the galloping acceleration is processed using the galloping amplitude monitoring method suggested in this study, the monitoring system’s galloping amplitude may be calculated. The galloping amplitude of the monitoring system is then compared to a laboratory-measured standard amplitude to determine the accuracy of the proposed galloping monitoring system algorithm. The experimental equipment for wire galloping is shown in Figure 13.

### 4.2. Comparison of Experimental Results

The designed gallop monitoring system’s amplitude measuring accuracy is assessed on the test mentioned. Let the proposed gallop monitoring system collect 1000 acceleration data points in the directions of the x, y, and z axes, respectively. The galloping amplitude data may be acquired by using the galloping amplitude algorithm proposed in this work to process acceleration data. The amplitude monitored by the monitoring system is compared to the galloping amplitude of the test rig to determine the accuracy of the amplitude measurement of the monitoring system. Because the influence of the *z*-axis in the direction parallel to the wire is not apparent, wire galloping focuses on the galloping displacement of the x and y axes in the direction of the wire cross-section. Next, we mainly analyze the galloping displacement in the *x* and *y*-axis directions.

#### 4.2.1. Acceleration Data Acquisition

**Figure 14:**
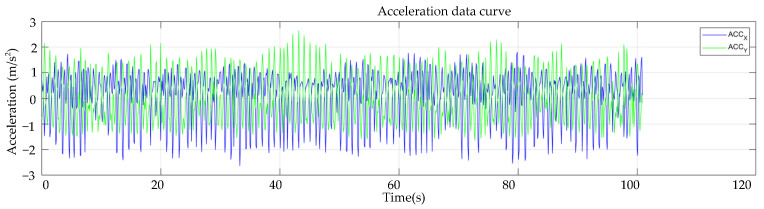
*x*-axis and *y*-axis acceleration data curve.

The x and y axes acceleration data curves are collected by the galloping monitoring system using Matlab software. The acceleration curve recorded by the galloping monitoring system is presented in Figure 14. From the acceleration curve in the picture, it is noticeable that the acceleration recorded by the monitoring system is a curve that varies regularly. The recorded acceleration curve is also a regular motion, as the galloping test rig has a constant-amplitude motion. The acceleration curve can be used to reflect the motion law of the galloping test bench to some extent.

#### 4.2.2. Galloping Algorithm Comparison

**Figure 15:**
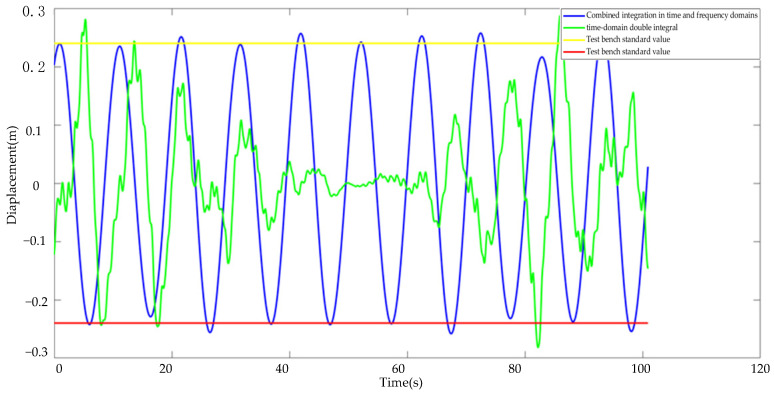
Algorithm curves of different gallop amplitudes.

Because the *x*-axis and *y*-axis integration procedures are identical, the galloping algorithms are compared using the *x*-axis as an example. The curve shown in Figure 15 includes the amplitude measured by the galloping amplitude monitoring algorithm developed in this article, the measured amplitude of the current least-squares technique and the time-domain double integration method, and the experimental standard value. As can be seen from the data curve, the galloping amplitude curve formed by the approach proposed in this study is nearly equal-amplitude motion, and the amplitude is closer to the laboratory standard value. Although the largest amplitude point that the existing algorithm can monitor is quite close to the normal laboratory value, the overall data curve differs significantly from the known constant amplitude motion in the laboratory. The method proposed in this research may successfully restore the test bench’s galloping features, both in terms of amplitude monitoring accuracy and the wire movement trend shown by the overall integral curve.

### 4.3. Validation of Experimental Results

#### 4.3.1. Gallop Amplitude Monitoring

**Figure 16:**
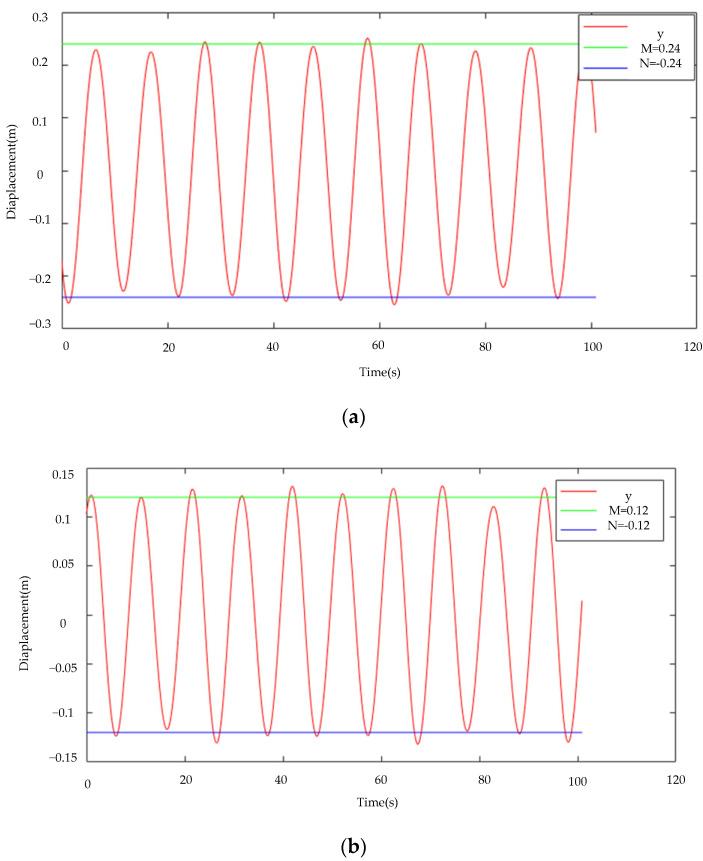
Galloping amplitude curve (**a**) *x*-axis displacement (**b**) *y*-axis displacement.

The motion of the galloping test rig is equal to amplitude motion, and the galloping amplitudes of the x and y axes monitored by the test rig are 0.24 m and 0.12 m, respectively. The gallop amplitude curves of the x and y axes tracked by the gallop amplitude monitoring system described in this research are displayed in Figure 16. It is clear to see from the figure that the amplitude curves monitored by the *x*-axis and *y*-axis oscillate up and down at 0.24 m and 0.12 m, respectively, and the motion is roughly equal-amplitude motion, and the maximum amplitude point error is within 10 percent. Through the amplitude error, it can be seen that the galloping amplitude of the wire can be better monitored.

#### 4.3.2. Galloping Track Restoration

**Figure 17:**
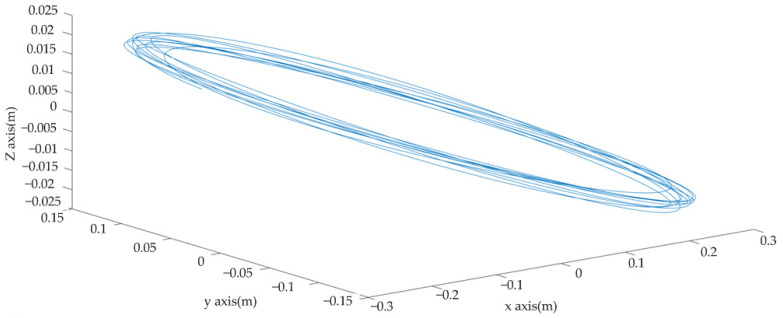
Wire galloping space trajectory diagram.

The monitored wire’s movement can be better described using the spatial galloping trajectory. In MATLAB software, the galloping amplitude of the x, y, and z axes is obtained using a galloping algorithm, and the spatial galloping trajectory of the wire is drawn. Figure 17 displays the spatial gallop trajectory map of the x, y, and z axes. It is clear to see from the trajectory diagram of the wire space that the wire performs nearly equal-amplitude elliptical movements in space. It can be observed from the spatial trajectory diagram that the spatial trajectory of the wire is consistent with the known movement trajectory of the wire on the test bench. The trajectory map can prove that the monitoring system can mimic the galloping course of the wire adequately.

#### 4.3.3. The Galloping Amplitude Residual Curve

**Figure 18:**
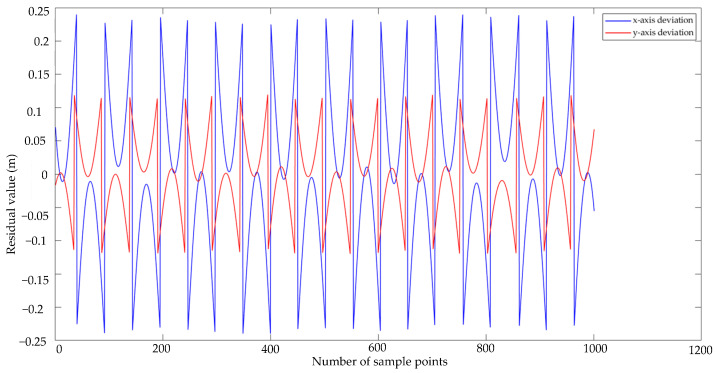
*x*-axis and *y*-axis deviation graph curves.

The galloping amplitude monitoring system calculates the difference between 1000 displacement data points acquired on the x and y axes and the standard displacement. The x and y axes deviation graph was drawn by using MATLAB software. Figure 18 depicts the deviation graph for the x and y axes. The deviation graph curve likewise exhibits periodic oscillations of identical magnitude, as can be observed from the deviation graph curve. The x and y axes had known input amplitudes of 240 and 120 mm, respectively, while the mean deviation values for the x and y axes were 0.642 mm and 0.084 mm, respectively. The deviation values are minimal when compared to the test rig’s known amplitudes. The average deviation value can demonstrate that the galloping amplitude monitoring system is fairly steady in its operation.

## 5. Conclusions

Based on an examination of existing transmission line galloping monitoring system demands, this paper proposes an intelligent transmission line galloping monitoring system. In order to address the challenges that exist in the installation of the monitoring terminal equipment of the current gallop monitoring system, this research proposes that a new device be installed on the original spacer bar device to solve the monitoring terminal installation problem. The impact on the original spacer bar is assessed in the structural design, and the effects of the improved spacer bar and the original spacer bar on the transmission line are compared and investigated. According to the simulation results, the error of the galloping displacement of the installed improved spacer bar is within 7.38 percent of that of the original spacer bar under the same wind field conditions. The improved spacer bar structure’s galloping displacement error may fulfill the original transmission line’s technical standards.

In the algorithm design of the galloping monitoring system, the problem of the fusion of gravity acceleration and measurement acceleration generated by the twisting phenomena of the wire galloping is not considered. It is proposed that the Kalman filter and the Mahony complementary filter be used to tackle the problem of erroneous acceleration measurement caused by wire twisting. The calculated attitude angle is used to remove the gravitational acceleration component caused by the wire twist from the measured acceleration. The comparison data for attitude angle computation shows that the algorithm proposed in this study can produce a more accurate attitude angle.

The least-square approach, adaptive smoothing method, and time-frequency domain hybrid integration algorithm are offered to tackle the effect of the vast trend term caused by the double integral in the time domain. Adaptive smoothing filtering is presented to better compute the smoothing order and remove the amplitude error caused by large or small smoothing filtering orders. The benefits of fewer trend terms generated by time-domain one-integral and less effect of low-frequency in frequency-domain one-integral are combined in a hybrid integration in the time-frequency domain to lessen the effects of other faults. When compared to the amplitude of the standard test bench, the amplitude measured by the monitoring system has an error of less than 10%. The monitoring system has restored a galloping trajectory that is nearly identical to that of the test bench. The galloping amplitude monitoring system proposed in this study satisfies actual engineering requirements.

According to the experimental results, the monitoring and standard amplitudes have a small error. In future research, potential error sources will be investigated in order to lessen the influence of errors on experimental outcomes.

## Figures and Tables

**Table 1:** Experimental results of attitude Angle.

Rotation Angle	EKF *x*-axis (°)	QUAT *x*-axis (°)	EKF *y*-axis (°)	QUAT *y*-axis (°)	EKF *z*-axis (°)	QUAT *z*-axis (°)
0°	0.2	0.3	0.16	0.36	0.45	0.56
30°	30.1	29.7	29.75	30.37	30.3	29.63
60°	59.81	60.32	59.9	60.25	60.62	59.26
90°	89.9	90.21	89.83	90.33	90.86	89.1

## Data Availability

Data is contained within the article.

## References

[1] Barcellona S., Piegari L. (2020). Effect of current on cycle aging of lithium ion batteries. J. Energy Storage.

[2] Patrizi N., LaTouf S.K., Tsiropoulou E.E., Papavassiliou S. (2022). Prosumer-Centric Self-Sustained Smart Grid Systems. IEEE Syst. J..

[3] Nigol O., Buchan P. (1981). Conductor galloping part I-Den Hartog mechanism. IEEE Trans. Power Appar. Syst..

[4] Quan Y.S., Zhou E., Chen G., Zhao X. (2013). New Methodology of On-Line Monitoring for Transmission Line Galloping. Adv. Mater. Res..

[5] Huang X., Huang G., Zhang Y. Designation of an on-line monitoring system of transmission line’s galloping. Proceedings of the 2009 9th International Conference on Electronic Measurement & Instruments.

[6] Zhao S.P., Zhao S.X., Zhang Y.P., Wang Y.H. (2021). Design and Data Processing of Galloping Online Monitoring System for Positive Feeder of High-Speed Railway Catenary in Gale Area. IEEJ Trans. Electr. Electron. Eng..

[7] Rui X., Huang H., Zhang S., Teng W. On-line monitoring system on power transmission line galloping based on fiber grating sensors. Proceedings of the 30th Chinese Control Conference.

[8] Bjerkan L. (2000). Application of fiber-optic Bragg grating sensors in monitoring environmental loads of overhead power transmission lines. Appl. Opt..

[9] Mahajan S.M., Singareddy U.M. (2012). A real-time conductor sag measurement system using a differential GPS. IEEE Trans. Power Deliv..

[10] Yang W., Shao Y., Lv Z., Yang X., Li Q., Xie K., Wei J., Zhang B. Study on monitoring for power transmission line galloping based on monocular vision method. Proceedings of the 2014 International Conference on Power System Technology.

[11] Li W.H., Tajbakhsh A., Rathbone C., Vashishtha Y. Image processing to automate condition assessment of overhead line components. Proceedings of the 2010 1st International Conference on Applied Robotics for the Power Industry.

[12] Huang X.B., Zhao L., Chen G.M. (2016). Design of a Wireless Sensor Module for Monitoring Conductor Galloping of Transmission Lines. Sensors.

[13] Zhang P., Chang J., Qu B., Zhao Q. (2016). Denoising and trend terms elimination algorithm of accelerometer signals. Math. Probl. Eng..

[14] Zhang A.B., Chen T.Y., Liu X.X., Zhang Y.J., Yang Y.T. (2015). Monitoring data filter and deformation information extraction based on wavelet filter and empirical mode decomposition. Appl. Mech. Mater..

[15] Li Z., Fang H., Ke X., Li F., Wang Y. (2019). Application of sliding average method to MEMS gyroscope signal trend extraction. J. Electron. Meas. Instrum..

[16] Cheng X., Zhou T., Sun K. (2019). Time/frequency-domain integration method of vibration acceleration signal processed by wavelet denoising. Power Energy.

[17] Chen Y., Li M., Chen P.P., Xia S.X. (2019). Survey of cross-technology communication for IoT heterogeneous devices. IET Commun..

[18] Zhang S., Ma W., Yu M., Zhang F., Chen J. (2021). Design of monitoring system for redundant communication power supply based on ZigBee. J. Phys. Conf. Ser..

[19] Huang X., Zhao L., Zhou K., Lou B., Li M., Zhang Y. (2014). Conductor galloping monitoring system based on inertial sensor for transmission lines. High Volt. Eng..

[20] Zhao G., Lu Z., Wang X., Peng Y., Chang S. (2022). Full Scale Experiment for Vibration Analysis of Ice-Coated Bundled-Conductor Transmission Lines. KSCE J. Civ. Eng..

[21] Keyhan H., McClure G., Habashi W.G. (2013). Dynamic analysis of an overhead transmission line subject to gusty wind loading predicted by wind-conductor interaction. Comput. Struct..

[22] Keutgen R., Lilien J.-L. (2000). Benchmark cases for galloping with results obtained from wind tunnel facilities validation of a finite element model. IEEE Trans. Power Deliv..

[23] Lou W.J., Lv J., Huang M.F., Yang L., Yan D. (2014). Aerodynamic force characteristics and galloping analysis of iced bundled conductors. Wind Struct..

[24] Lu J.Z., Wang Q., Wang L.M., Mei H.W., Yang L., Xu X.J., Li L. (2019). Study on wind tunnel test and galloping of iced quad bundle conductor. Cold Reg. Sci. Tech..

[25] Liu J., Yan B., Mou Z., Gao Y., Niu G., Li X. (2022). Numerical study of aeolian vibration characteristics and fatigue life estimation of transmission conductors. PLoS ONE.

[26] Nigol O., Buchan P. (1981). Conductor galloping-Part II torsional mechanism. IEEE Trans. Power Appar. Syst..

[27] Huang G.Z., Yan B., Liu J.Q., Wu C.A., Lv Z.B. (2020). Experimental Study on Torsional Behavior of Twin Bundle Conductor Lines. IEEE Trans. Power Deliv..

[28] Liu M., Cai Y.L., Zhang L.H., Wang Y.Q. (2021). Attitude Estimation Algorithm of Portable Mobile Robot Based on Complementary Filter. Micromachines.

[29] Phan M.Q., Vicario F., Longman R.W., Betti R. (2018). State-Space Model and Kalman Filter Gain Identification by a Kalman Filter of a Kalman Filter. J. Dyn. Syst. Meas. Control-Trans. ASME.

[30] Liu H., Hu F., Su J.S., Wei X.W., Qin R.S. (2020). Comparisons on Kalman-Filter-Based Dynamic State Estimation Algorithms of Power Systems. IEEE Access.

[31] Gui H.C., de Ruiter A.H.J. (2018). Quaternion Invariant Extended Kalman Filtering for Spacecraft Attitude Estimation. J. Guid. Control Dyn..

[32] Wang G., Xue R. (2019). Quaternion Filtering Based on Quaternion Involutions and its Application in Signal Processing. IEEE Access.

[33] Bai F., Liu Y., Liu Y., Sun K., Bhatt N., Del Rosso A., Farantatos E., Wang X. (2015). Measurement-based correlation approach for power system dynamic response estimation. IET Gener. Transm. Distrib..

